# Enhancing motivation within a rapid opioid substitution treatment feasibility RCT: a nested qualitative study

**DOI:** 10.1186/1747-597X-9-44

**Published:** 2014-11-18

**Authors:** Rachel Ayres, Jenny Ingram, Avril Rees, Jane Neale, Angela Beattie, Maggie Telfer

**Affiliations:** Bristol Drugs Project, Brunswick Square, Bristol, BS2 8PE UK; School of Social and Community Medicine, University of Bristol, Oakfield House, Bristol, BS8 2BN UK; School of Social and Community Medicine, University of Bristol, Canynge Hall, Bristol, BS8 2PS UK

**Keywords:** Opioid substitution treatment, Motivational interviewing, Cognitive dissonance, Self-determination theory, Qualitative interviews

## Abstract

**Background:**

Opioid substitution treatment (OST) has multiple benefits for heroin injectors and is an evidence-based major component of international treatment. The current qualitative study sought to explore participants’ attitudes to and reasons for participating in a feasibility randomised trial in primary care offering ‘same day’ OST (methadone) for injecting heroin users compared to usual care.

**Methods:**

Twenty injecting heroin users (8 intervention and 12 controls; 16 males and 4 females) were interviewed; purposive sampling was used to select a maximum variation sample from those who agreed; and analysis used thematic methods.

**Results:**

Motivation to join the trial included the need to secure treatment set against some ambivalence due to previous negative experiences of trying to obtain OST. Positive effects of securing methadone via the trial, included self-reported improvements in health and self-care; reduction in crime, stress and drug use. Completing the baseline questionnaires at recruitment appeared to enhance motivation for treatment for all participants. For some control participants, this motivation seemed to increase a sense of self-efficacy and cognitive dissonance generated was resolved by seeking treatment from their GP. Self-determination theory suggests that behaviour change may have been initiated during the recruitment appointment, resulting in an increased determination to seek treatment amongst control participants.

**Conclusions:**

Taking part in the ‘script in a day’ trial enabled participants in the intervention arm to gain same-day access to methadone and reduce their drug use. For those in the control arm, completing the baseline questionnaires at recruitment appeared to create cognitive dissonance between their current health state and own aspirations, so increasing motivation for treatment. Over 50% obtained and were still in receipt of OST (methadone or buprenorphine) at the 3 month follow-up. We suggest that a regular ‘health evaluation’ for injecting heroin users not in treatment, paired with low-barrier access to treatment, may be a way of exploring this and encouraging more into obtaining OST more quickly and at the best time for them. This intervention should be delivered without pressure for change.

**Clinical trial registration:**

This trial is registered with International Standard Randomised Controlled Trial Number Register: SCript In a Day for injecting drug users: feasibility trial: ISRCTN16846554.

## Background

Opioid substitution treatment (OST) has a strong evidence base, is a major component of current treatment in many countries, and has multiple benefits for heroin injectors [1–3]. OST is key to reducing HIV and Hepatitis C virus prevalence amongst injecting drug users (IDU’s) [4, 5]. Interventions to increase the uptake, or to re-engage with OST, are routine components of harm reduction within needle syringe programme (NSP) environments [5, 6]. Neale’s studies suggest that increased engagement with OST programmes could be achieved by improving service delivery, staff communication and attitudes, but also that IDUs need to be motivated and feel positive about seeking help for their drug use [7].

This position is set amongst a growing body of literature which sees OST through a variety of lenses: from a bio-medical treatment model to socio-cultural control of deviance model [8, 9]. There is also tension between the harm reduction focus of entering OST and the current ‘Recovery Agenda’ [1]. This underpins national government drug policy as implemented by Public Health England. It has been suggested that this agenda could move injecting drug users into detoxification and abstinence before they are ready and so perpetuate cycles of problematic drug use [10].

OST (methadone and buprenorphine) is readily available via Primary Care in Bristol, UK, with over 90% of health centres participating in a Shared Care scheme with staff from Bristol Drugs Project (BDP). Average waiting times for OST at the time of this trial were approximately 1 week between GP appointment and start of prescribing. Despite this relative ease of access, 20-25% of NSP users were not on OST at any one time (BDP unpublished data 2007–2011), citing difficulties in getting GP appointments and waiting lists as barriers, meaning that windows of motivation were missed and ambivalence returned. Participants in focus groups carried out during 2008/2009 asked for access to same-day methadone, whereby motivation for treatment could be harnessed immediately and some of the real or perceived barriers reduced (BDP unpublished data).

Motivational interviewing (MI) has long been the tool of choice in substance misuse services; alongside cognitive behavioural therapy, and group or individual skills training [11]. Systematic reviews of the evidence base for MI reveal that MI is: particularly effective in enhancing entry into drug treatment [12]; can reduce the extent of substance abuse compared to no intervention, [13]); and that MI and case management approaches to increase the uptake of injecting drug users into treatment were both promising but the evidence inconclusive [14]. Markland et al. [15] note that MI is widely used to promote behaviour change, but lacks a coherent theoretical framework for understanding its processes and efficacy. They propose that self–determination theory (SDT) can offer a broad framework for studying and understanding motivation for change [15].

We have used these models to help understand the outcomes from a feasibility RCT (‘Script in a Day’ trial) which offered a ‘same day’ OST (methadone only) prescription for injecting heroin users compared to usual care. Outcomes showed little difference between the intervention and usual care groups in terms of retention on OST at 3 months (Beattie et al. personal communication). ‘Usual care’ in the city was an appointment with their GP followed by a wait for urinalysis and a ‘shared-care’ drugs worker to support prescribing. The trial included this nested qualitative study in which we sought to explore the trial processes, understand the effects of and acceptability of the intervention and outcomes for control participants. This paper focuses particularly on understanding the positive outcomes and motivation for change amongst a number of control group participants, offering motivational interviewing and self-determination theory [15] as frameworks for understanding their positive behaviour change following recruitment to the trial.

## Methods

Adult participants were recruited to the trial through the NSP at Bristol Drugs Project (http://www.bdp.org.uk) between October 2011 and September 2012 and if they agreed to take part, validated questionnaires were completed: the Treatment Outcomes Profile Measure (TOPS) [16], widely used in drug services, measuring substance use, injecting risk behaviour, crime, health & social functioning; the EQ-5D [17], a health related quality of life and utility status measure; and SF-12 a generic health status measure providing both mental component summary and physical component summary scores[18]. Participants were then randomised into the intervention arm (same day appointment with the intervention GP to obtain a three-week course of methadone followed by transfer back to their own GP for on-going care) or control group (advice about how to contact their own GP to obtain treatment). The day after recruitment they could return for a payment of £15 to cover their time involved in the recruitment process.

At the three month follow-up all participants in the trial were invited to be interviewed for this qualitative nested study. A purposive sample was selected from those who agreed to include both trial arms, males and females, and a range of ages. The semi-structured interviews were framed around a topic guide developed from the literature and discussions between the qualitative team and the Harm Reduction workers at BDP. Interviews were conducted at BDP and took around 30 minutes each exploring participants’ attitudes to the trial, reasons for joining the trial and the impact of randomisation to either arm. Participants were paid £15 for their time involved in the interview.

Interviews were carried out by three Harm Reduction workers from Bristol Drugs Project, trained to be recruiters and interviewers for the trial. Data collection and analysis were conducted in parallel and interviews continued until data saturation was reached with no new themes arising from the data [19]. This was an iterative process and towards the end of sampling, greater emphasis was given to choosing control arm participants, as early interviews had revealed a level of motivation to seek and secure treatment, which seemed to have been generated by the recruitment appointment itself. Preliminary findings from early interviews were framed in additional questions which were then explored in the later interviews with different participants. Interviews were audio-recorded, transcribed verbatim, anonymized and coded through repeated reading of the transcripts [20]. Four authors (RA, JI, AR, JN) coded interviews and various combinations of two authors double coded them all. Thematic analysis using an inductive approach [21] was used to scrutinise the data to identify and analyse patterns, themes and sub-themes across the dataset. Themes and any discrepancies were discussed extensively with the qualitative researcher (JI) guiding the process to achieve a coding consensus and ensure robust analysis.

Ethics approval was given by National Research Ethics Service, South West (11/H0102/1).

## Results

Three hundred and eleven injecting drug users who attended the needle exchange during the recruitment period were eligible to join the trial, by reporting that they were not in receipt of opioid substitution treatment nor had been during the previous two weeks. One hundred of these consented to take part. At the 3-month outcome, follow-up data were available for 96 participants (85 from face-to-face contacts, 11 from medical records only); 25/48 (52%) in the intervention arm were on OST and 24/47 (51%) of the control arm. Sixty of those seen face-to-face agreed to be interviewed for the qualitative study, and 20 of these were selected for an interview, eight from the intervention arm and 12 controls; 16 males and four females. This gender mix was representative of the overall trial composition. Their mean age was 38 years (range 22 to 53 years) and the females were slightly younger (mean age 32 years) compared to the males (mean age 39 years old). Before taking part in the ‘Script in a Day’ trial, participants had not been on OST (methadone or buprenorphine) for a median time of 27 weeks, which perhaps showed some ambivalence to obtaining OST in trial participants, given the relative ease of obtaining OST in Bristol at the time.

Themes from the interviews emerged around motivation to join the trial (starting the journey to recovery and ambivalence), responses to being randomised from the intervention (positive) and control (disappointment) participants, and enhancing motivation for treatment at recruitment (developing self-efficacy and resolving cognitive dissonance).

### Motivation to join the trial

#### Starting the journey to recovery

Reasons cited for joining the trial were primarily a desire to get on OST. Some were motivated for harm reduction purposes and others as a step to detoxification and rehabilitation, which they saw as starting their journey to recovery:

“I was in a very bad place and was desperate for something to stabilize me” (#306; Male; 27 y; control).

“I was struggling to inject myself….struggling to find a vein…in the end it would congeal in the pin and I’d have to squirt it away … so that’s why when I heard about (the trial), that’s why it came at the right time” (#218; Male; 40 y; intervention).

“It was the first step …getting back into recovery…To get into detox I had to be on 30 mls (Methadone) maximum. I knew I was on borrowed time. I knew…that it wouldn’t be long before I was arrested again for what I was doing to get my drugs” (#127; Male; 50 y; intervention).

Some participants were already in the process of securing OST and hoped to speed up the process by joining the trial:

“I know it can take a long time to get a script through Shared Care.” “I really do (did) need to go and get a script anyway, so I did. I went and signed in with the doctor” (#011; Male; 35 y; control).

“I was desperate at the time, so I had to try all channels that may have medicated me as quick as possible…I think I done the test (trial recruitment) on the Friday and my appointment (for scripting) was the following Tuesday anyway so…” (#315; Male; 33 y; control).

#### Ambivalence and previous negative experiences

Most interviewees had either had negative experiences of getting OST in the past or assumed that it would be difficult in the future and this had become a barrier to seeking treatment from their GP in primary care or any other secondary care agency. A same-day prescription was therefore an attractive option:

“I’d been thinking about getting scripts myself, but the process seemed long and daunting…that ‘in-a-day’ thing caught my attention, I thought “yeah” (#106; Female; 33 y; intervention).

“Even if you know where to go (i.e. to the GP) there’s this thing of like, you know, er “phew they ain’t gonna be interested in sorting me out, d’you know?” (#310; Male; 40 y; control).

Despite relatively short waiting times for OST in Bristol, any wait can feel too long and may result in a loss of motivation and missed appointments. Participants talked about length of time to book a GP appointment, needing to provide a urine sample and returning for a second appointment and prescribing. For some, prescribing could not start until a shared care worker was also available.

“Several times I have made doctor’s appointments but when the day has come I’ve been alright that day so I’ve not bothered” (#218; Male; 40 y; intervention).

“It takes forever to get a script, so (when) you said ‘script-in-a-day’, obviously I took an interest…” (#009; Male; 46 y; intervention).

“The last time I got a script through my doctor it took ages, …I think it took about three weeks, so it was like a joke” (#108; Male; 30 y; intervention).

A minority of health centres in Bristol do have long waiting lists and one or two still do not provide OST for opiate users:

“I’d had problems trying to get scripted at XX health centre, even though I lived in the area there was a long waiting list” (#306; Male; 27 y; control).

“The trial for me was beneficial. Because my – my doctor’s surgery, like my normal GP, doesn’t prescribe methadone” (#127; Male; 50 y; intervention).

### Intervention participants’ responses to randomisation

A very positive response was expressed by all eight interviewees who were randomised into the intervention arm.

“For it to actually happen (get a same day script) was brilliant” (#218; Male; 40 y).

“Fantastic. So quickly, it was fantastic, instead of having to wait …” (#101; Female; 36 y).

#### Positive impacts of securing same-day methadone

The same-day prescription resulted in immediate self-reported improvements in several domains.

Crime reduction: removing the need to commit crime to fund a habit gave time to concentrate on improving health and relationships.

“It turned my life around and in a short space of time I didn’t have to go out and commit crime to fund, to get drugs (#108; Male; 30 y).

“I could go to sleep thinking “it doesn’t make any difference at all if I wake up with no money at all” I know that in the morning when I wake up all I’ve got to do is walk round to the chemist and I’m ok for the day and I don’t have to risk my liberty…to fund” (#218; Male; 40 y).

Health improvements, reduction in stress and in drug use:

“I’m not injecting myself left right and centre … you don’t see me down here (*at the NSP*) anymore very often … its just a hell of a lot better … 100% better. I’m eating better, everything” (#009; Male; 46 y).

“I haven’t gotta worry about waking up in the morning feeling ill, not wanting to come out, feeling lethargic, feeling irritable. Relationship-wise is just absolutely brilliant, absolutely great” (#219; Female; 28y).

“Through getting the script … it was the biggest stress that I had going on in my life, taken care of” (#109; Male; 41 y).

“It takes the amount you’re using down … I don’t have to use it (heroin) every day…from using a gram a day to sometimes maybe a bag a day” (#106; Female; 33 y).

A step to independence, increased sociability and self-care:

“I think it’s fantastic. Without it I wouldn’t be clean today and doing what I’m doing and getting on with my life (#101; Female; 36 y).

“It was my first step into … getting back into recovery…that’s the sort of way in for a lot of people, then other work can start, the work to start changing behaviours and lifestyles and things like that” (#109; Male; 41 y).

The participants’ experience of getting a same-day prescription was reported as both a positive and straight forward process:

“It was tremendous I was blown over…I timed from when I first… took part in the trial…contacting the surgery, arranging an appointment, seeing the doctor, being prescribed, the whole thing was just under…two hours (#109; Male; 41 y).

“Within half an hour I got it, picked up my methadone from the chemist and then I was on daily pick up” (#218; Male; 40 y).

However, one participant reported the negative impact she felt after her need to use heroin had been addressed via methadone, where loss of routine and regular contact with using associates left a void

“Normally I start my day at 11 o’clock, make money and have a hit. Then I got out of bed and I’d think “what do I do?” I didn’t see the friend I normally see to associate with. I suddenly felt all in a big space not knowing what to do with it really, so it was a bit daunting”…it was a big thing feeling “what do I do now” (#106; Female; 33 y).

### Control participants’ responses to randomisation

#### Disappointment and negative thoughts

The response to being randomised to the control arm was universally disappointing. Whether or not the individual was ambivalent at the point of recruitment, desire for treatment grew during the recruitment appointment when health questionnaires and drug audits were completed and responses were strongly expressed:

“It was pretty heart breaking when I got the control group” (#306; Male; 27 y).

“Yeah I was devastated” “I knew that I had to go back out and buy street drugs, and I did not want to, I really didn’t. I’m sick and tired doing street drugs” (#219; Female; 28 y).

“Gutted” “yeah really, really disappointed, yeah.” “…Yeah sort of when you take a stab at something and you get knocked back it sort of makes you think, Phew, why did I bother in the first place?” (#122; Female; 29 y).

“…they said “now you’ve got to get a GP, you’ve got to get a drugs worker” and all, so it all seems like a big hill, d’you know?… when you’re out there as an addict on the street, it’s er getting it together to do these normal things, I know it doesn’t sound a lot, but it’s er hard work staying on top of things” (#310; Male; 40 y).

“I knew I wouldn’t be able to get a script quickly elsewhere…” (#011; Male; 35 y).

Only two interviewees from the control group were phlegmatic about their outcome:

“I know (knew) I need to (register with a GP) but its finding one that will take you on when you are NFA (*no fixed abode*) and all the rest of it” (#310; Male; 40 y).

“I thought to myself - at the end of the day, at least someone had taken the time and effort to try and help me on the road to getting a script” (#006; Male; 22 y).

### Enhancing motivation for treatment at recruitment

#### The role of recruitment

Early qualitative interviews indicated that participants may have experienced a shift from ambivalence towards motivation for treatment during the trial recruitment appointment itself and in particular through completing the self-evaluation questionnaires. This may partly explain the extreme disappointment expressed by most of those recruited to the control arm.

“It made me think a lot actually about myself…I don’t actually sit down and think about my overall day, usage or anything like that. But when I was going through the questionnaire I actually did think about “God … things aren’t quite as good as I thought they were” (#219; Female; 28 y; control).

“It felt a bit personal, answering some of the questions, .. I thought, it’s all for a good cause, it’s helping me in the long run, these are some things I haven’t even asked myself about myself… I went home, it gave me a bit of time to reflect on my er own personal experience a bit more deeply that I usually would” (#006; Male; 22 y; control).

#### Developing self-efficacy

Drug users can experience a great deal of pressure to be in treatment (including from family and friends, the criminal justice system, primary health and specialist services) which can provoke resistance or undermine self- efficacy:

“..previously I’ve had scripts almost forced on me…through like er drug treatment testing orders or drug rehabilitation requirements, whatever, and I carried on using street drugs,” (#310; Male; 40 y).

Opting to participate in the ‘script in a day’ trial seemed to offer a sense of autonomy:

“..came in just because the trial was going on to be honest with you…it gave me the er – the push that I think I needed…..it put something in my head… well alright, you know, it didn’t happen (getting the same-day script), ..but it’s not the end of the world. You know, you can make it happen if you chose, and that’s what I did you know” (#007; Male; 53 y).

“I went home it gave me a bit of time to reflect on my er own personal experiences a bit more deeply than I usually would.” “It’s helped motivate me into realising that I can do it myself and I don’t need outside support all the time.” (#006; Male; 22 y).

#### Resolving dissonance

Having experienced an increase in motivation and then subsequent disappointment at being randomised to the control arm, 24/47 participants (51%) went on to secure OST (methadone or buprenorphine) within the three month trial period and six of these were interviewed:

“.. once they said I was in the control group then I thought, you know, “I really do need to go and get a script anyway”, so I did, I went and signed in with the doctor…” (#011; Male; 35 y).

“I suppose it made me get off me bum and go and get a script somewhere else” (#002; Male; 43 y).

#### Changing/adding cognitions

Those recruited to the control arm who didn’t secure OST within the three month follow-up period cited various barriers. Two recruits talked about long waiting times and changes in motivation; neither had actually tried to make appointments.

“…with the doctors I’m with, you’ve gotta wait weeks for an appointment with a normal doctor, and then weeks again for an appointment with a doctor that can help you with a medicated script. So you’re looking at a month, maybe even more. And, you know, if you’re using that whole entire month, the likelihood is you ain’t gonna make those appointments, and that’s been the case with me really” (#122; Female; 29 y).

“It’s quite hard to get an appointment ‘cos you’ve gotta ring first thing in the morning, and if you haven’t got credit and that, you can’t get one basically, and there’s only certain doctors that deal with scripts and er – in this area – and there’s a lot of people that are on drugs, and they take up all the appointments straight away” (#114; Male; 35 y).

Another had made two GP appointments but missed them:

“I tried to get a script through him (GP) but I was missing appointments and things like that, so I never got the script” (#015; Male; 48 y).

One participant talked about a previous positive experience with buprenorphine and cited a reluctance to start back on methadone as a barrier, despite being disappointed at being randomised to the control arm:

“I wanted to be back on the subu (buprenorphine), so it was taking a step back, I thought by going back onto Methadone” (#143; Male; 44 y).

## Discussion

The feasibility trial found that there were no differences between the intervention and control groups in terms of numbers who were still on OST (methadone or buprenorphine) at three months. This nested qualitative study has shown how taking part in the trial enabled several in the intervention arm to secure OST, detoxify and achieve freedom from their problematic drug use. For others, remaining on OST reduced their injecting drug use and allowed for the building of ‘recovery capital’ [1]. A small number of intervention arm recruits were no longer on OST at three months. One intervention group interviewee illuminated the risk of providing a pharmacological intervention (methadone) in the absence of psycho-social support, where the sudden absence of a drug-using routine and associated friendships, left a destabilising void. This perhaps adds to the current dialogue about potential risks associated with early entry into ‘Recovery’ [10], driven by the Recovery Agenda [1].

All recruits to the trial said they were ‘interested’ in securing OST but, despite the relative ease of access to OST in Bristol, had faced barriers (real or perceived) and had not been able or motivated to do so in the period prior to recruitment. For those in the control arm, completing the baseline questionnaires appeared to create some cognitive dissonance between their current health state and their personal aspirations, which was not immediately resolved by being offered treatment within the trial.

All control arm participants expressed disappointment at not being in the intervention arm. However, over 50% went on to secure OST. We offer the following explanation of why this group of individuals may have chosen to seek treatment independently. Cognitive dissonance theory is founded on the assumption that individuals seek consistency between their expectations and their reality and will strive to reduce the discomfort that any discrepancy between the two causes [22]. Exploring this in terms of self-determination theory may begin to explain why many of those in the control arm had better outcomes than expected.

Motivation for change builds when individuals start to perceive discrepancy between current and desired behaviour (ambivalence) and then options to resolve the cognitive dissonance experience are identified [23]. This is usually the first, (directive) step in Motivational Interviewing (MI) for eliciting behaviour change, a technique with an established evidence base in the substance misuse field [24, 13]. In the context of recruitment to this trial, there was no premeditated, attempt by the interviewers to develop a discrepancy with the intention of eliciting behaviour change. However, we postulate that carrying out a self-evaluation of health and social wellbeing through the baseline questionnaires at the recruitment appointment, provoked cognitive dissonance [22] between the participants’ *status quo* and their core values or aspirations; one of the first stages in an MI intervention.

Festinger [22] described the necessity to resolve ‘dissonance related discomfort’ quickly. Whilst the autonomic discomfort of enhanced dissonance is thought to be very short-lived (minutes) its effects may last up to two weeks [25]. It may be that some of those recruited to the control arm of the trial, resolved their dissonance by seeking and securing treatment within a short period following recruitment to the trial (mostly within 2 weeks). In others, dissonance may have been reduced by changing their cognitions to fit their behaviour and justifying on-going drug use, due to external pressures (such as difficulties in getting GP appointments) or by shifting emphasis or responsibility, perhaps by minimising the impact of their drug use or wanting something that was not available via the trial, such as a prescription for buprenorphine instead of methadone. Self-Determination Theory (SDT) [26] may further illuminate the drive to seek treatment that was seen after recruitment to the control arm. SDT suggests that individuals have innate tendencies towards personal growth and wellbeing and Cognitive Evaluation Theory (a sub-theory within SDT) specifies three fundamental psychological needs that must be facilitated for this to happen. These are: competence (facilitated when individuals are helped to develop clear expectations about what behaviour change could do for them), autonomy (pressure to engage in specific behaviours is minimised and individuals are encouraged to take action themselves, recognising that they have a choice regarding their behaviour) and connectedness (from supportive families or social networks, but may also be facilitated by supportive and respectful professional relationships) [27]. This model of understanding motivation for change through SDT is explored by Markland et al. [15].

We suggest that by choosing to participate in this trial, completing a self- evaluation of drug and alcohol use and quality of life questionnaires, conditions that supported change were created. Firstly, cognitive dissonance occurred as the participant described a picture of themselves at recruitment. A sense of competency, as described above, may have grown as the trial recruitment questionnaires illuminated broad areas of physical, emotional and social wellbeing that might benefit from behaviour change through access to OST. The route into treatment for drug dependency is well known in Bristol and as participants randomised to the control arm were given information about how to get OST via their GP (usual treatment) it may be that, for some of them, a sense of autonomy was fostered alongside a heightened desire for change. Many recruits may have had experience of mandated or coerced treatment via the criminal justice system, or pressure for treatment from concerned others (families and friends) or treatment agencies. For the recruitment appointment, the researchers were trained to adopt an impartial position and this attitude to treatment from research staff may have reduced external pressure and increased a sense of autonomy and internal motivation. The connectedness component is less clear, but we could speculate that time spent with researchers in a supportive but impartial environment added in a small way to this sense of feeling ‘related’ or ‘connected positively’ with others. In SDT terms, autonomy is seen as a continuum “reflecting the extent to which the person fully endorses and is committed to what they are doing” [15]. This moment of recruitment to the control arm may have pushed some individuals further along the continuum from external to a more self-determined form of regulation. Thus the valued outcome of behaviour change may have been indirectly highlighted during recruitment, alongside an enhanced sense of self-efficacy, resulting in an increased determination to seek OST.

### Strengths and limitations

The number of interviews in this study was relatively small but saturation was reached and no new themes were emerging from the data by the end of the interviews. The strengths of this study included the use of Harm Reduction workers, who were known to the participants, to conduct the interviews and this increased the participants’ confidence and enabled them to talk freely. The workers were also an intricate part of the analysis which helped the researchers to gain insights into the meanings of the interviews. However the use of ‘known’ researchers for the interviews may also have influenced what participants disclosed about the impact of their drug use, either exaggerating or minimising the impact, if it was felt that this might influence the researchers’ view of them. The qualitative interviews did not seek to gain insight into how dissonance was resolved. Our focus in this paper has been to offer a model that might explain why so many control arm participants went on to secure OST. We recognise that we have not attempted to explain why others in the control arm chose not to pursue treatment.

### Implications for practice

Incentivising service users to participate in Harm Reduction programmes is well established. Accepting that current UK drug treatment policy is to encourage injecting heroin users into OST as quickly as possible, this study illuminates a useful intervention that could be rolled out in NSP environments. We suggest that a regular ‘health evaluation’ for injecting drug users is carried out, similar to the alcohol brief intervention tool, AUDIT [28, 29], a self-completion questionnaire which can develop a discrepancy between drinking behaviour and view of self, raise anxiety and result in changes in alcohol use [30]. This ‘health evaluation’ tool could be offered to injecting drug users who are not currently in treatment with incentives for participation. Such a mechanism for encouraging OST uptake should be delivered without pressure for change and paired with increased access to low-threshold and evidence-based treatment.

## Conclusions

Taking part in the trial enabled participants in the ‘script in a day’ intervention arm to gain same-day access to OST and reduction in their drug use. For those in the control arm, completing the baseline questionnaires appeared to create cognitive dissonance between their current health state and own aspirations, so increasing motivation for and access to treatment. Many obtained and were still in receipt of OST at the 3 month follow-up.
